# The bud midge *Prodiplosis longifila*: Damage characteristics, potential distribution and presence on a new crop host in Colombia

**DOI:** 10.1186/s40064-015-0987-6

**Published:** 2015-04-30

**Authors:** Luis M Hernandez, Yoan C Guzman, Adriana Martínez-Arias, Maria R Manzano, John J Selvaraj

**Affiliations:** Departamento de Ciencias Agrícolas, Facultad de Ciencias Agropecuarias, Universidad Nacional de Colombia sede Palmira, Palmira, Valle del Cauca Colombia; Department of Agricultural Sciences, School of Agricultural Sciences, National University of Colombia at Palmira, Palmira, Valle del Cauca Colombia; Departamento de Ingeniería, Facultad de Ingeniería y Administración, Universidad Nacional de Colombia sede Palmira, Palmira, Valle del Cauca Colombia; Department of Engineering, School of Engineering and Management, National University of Colombia at Palmira, Palmira, Valle del Cauca Colombia

**Keywords:** Bud midge, Ecological niche, Plant damage, *Citrus*, *Solanum*, *Capsicum*

## Abstract

The Dipteran *Prodiplosis longifila* is a severe pest, mainly of Solanaceae, in South America and some years ago it damaged Tahiti lime crops in the United States. It is a potential invasive pest. Despite its presence in Colombia, nothing is known regarding the taxonomic identification of *P. longifila* or the characteristics of the damage it produces. Moreover, the current and potential distributions of this pest are unknown. To determine these factors, *P. longifila* was sampled in several Solanaceae- and *Citrus*^x^*latifolia* (Tahiti lime)-producing areas in Colombia. The larvae consumed tender foliage, flowers and fruits in tomato, fruits in sweet pepper, and buds in Tahiti lime. *P. longifila* was not found in asparagus or in potatoes. Its presence in Tahiti lime was previously unknown in Colombia. Adults recovered in the laboratory were taxonomically identified using male morphological characteristics such as the shapes of the genitalia, antenna and wing. *P. longifila* was found in the Andean region of Colombia. The ecological niche model for populations found in tomato suggests that *P. longifila* is limited in its distribution by altitude and variables associated with temperature and precipitation. The highest probability of occurrence is in areas where tomato, sweet pepper and the new host, Tahiti lime, are grown. Therefore, it is necessary to implement preventive measures, such as planting tomato materials free of *P. longifila* larvae, in areas where the pest is not yet present but where there is the potential for its development.

## Background

The bud midge *Prodiplosis longifila* Gagné is one of the most important pests of Solanaceae and asparagus in the Neotropics. In tomato, it can cause up to 100% loss in Colombia and up to 60% loss in Ecuador (Valarezo et al. 2003). In Peru, it causes considerable losses in crops of asparagus (*Asparagus officinalis* L; Cedano and Cubas 2012) and potato (*Solanum tuberosum* L.), where an infestation reaches up to 16% of buds (Kroschel et al. 2012). In the United States (Florida), it affected up to 25% of flower buds in Tahiti lime (*Citrus*^x^*latifolia* Tanaka ex Q. Jimenez) in 1984 (Peña et al. 1987). *P. longifila* has also been reported in pepper (*Capsicum frutescens* L.), sweet pepper (*Capsicum annuum* L.) and other companion plants (Gagné 1994; Gagné and Jaschhof 2014).

The larvae of *P. longifila* scrape the epidermal tissues of plant structures using piercing-sucking mouthparts (Gagné 1994). After undergoing three larval stages, the larvae pupate in the ground (Peña et al. 1989). Because larvae are small (<1.9 mm, Peña et al. 1989) and the crepuscular habits of adults make them difficult to find in the field, it is unclear what type of damage *P. longifila* causes. Our first objective was to characterise the damage caused by *P. longifila* in host plants. In Colombia, *P. longifila* feeds on foliar buds, flowers and fruits in tomato, whereas in the United States, it consumes the ovaries, stamens and pistils of Tahiti lime flower buds, causing their abortion (Peña et al. 1989). Based on the phytochemical distance of these two host plants, our second objective was to determine whether *P. longifila* of tomato is a morphologically different species from that reported in Tahiti lime in the United States. Because Colombia produces 92,304 tons of limes annually, representing 20.1% of all national (Ministerio de Agricultura y Desarrollo Rural 2006) and 0.09% of world lemon and lime production (FAO 2012), and the market for Tahiti lime is expanding in Europe and the United States (Ministerio de Agricultura y Desarrollo Rural 2006; Aguilar-Niño et al. 2012), our third objective was to determine if *P. longifila* is also found in Tahiti lime in Colombia.

Regarding the distribution of *P. longifila* in Colombia, this species was initially reported as a pest in the Valle del Cauca and the coffee region (Mena 2012), but its spread has expanded with concern to other regions of the country. Its current distribution is unknown. Risk maps based on the prediction of the climate distribution of a species are a management tool that allows the definition of current and future management areas of the insect pest (Ellsbury et al. 1999). These maps can be constructed based on the relationship of the species with different environmental factors (bio-variables) within its habitat (Guisan and Thuiller 2005). These maps can serve as ecological niche models (Peterson 2003) that allow the modelling of the invasion potential of the species (Mata et al. 2010). Our final objective was to determine the current and potential spatial distribution of *P. longifila* in tomato crops in Colombia, the main vegetable crop in the country. Tomato crops are planted in approximately 8,383 ha (8% of which is grown in the Valle del Cauca) that produce approximately 259,104 t/year with an average yield of 38.2 t/ha per cropping season (DANE 2012).

*Prodiplosis longifila* larvae consume leaf buds, flowers (ovaries and stamens) and small fruits of tomato plants, fruits of sweet pepper and flower buds and flowers of Tahiti lime. This is the first report of the insect consuming Tahiti lime (flower buds) in Colombia. *P. longifila* was found between 739 and 2168 m.a.s.l in The Andes of Colombia and it is limited in its distribution by altitude and variables associated with temperature and precipitation.

## Results

### Presence of *P. longifila* in sampled crops

During the study, 167 batches of crops of peppers, asparagus, Tahiti lime, potato, paprika and tomato were sampled in Colombia, and one batch of Tahiti lime was sampled in the United States. Of the 167 plots sampled in Colombia, 28% were located in the Valle del Cauca, followed by Boyacá (16%), Antioquia (15%), Huila (13%), Santander (9%), Caldas (5%), Cundinamarca (4%), Cauca (3%), Nariño (3%), Quindío (2%) and, finally, Risaralda (2%). As for the presence/absence of Cecidomyiidae, it was found that in Colombia, 53% of the crops visited presented at least one species. In the tomato crop, it was found that 65% of the sites visited had *P. longifila* infestation, while in sweet pepper and Tahiti lime, *P. longifila* was found in 31% and 75% of the crops visited, respectively. *P. longifila* was not found in crops of hot peppers, potatoes or asparagus. The morphological identification determined that the adult from tomato, paprika and Tahiti lime are morphologically similar and correspond to *P. longifila*.

### Geographical distribution and morphological description of *P. longifila*

**Figure 1:**
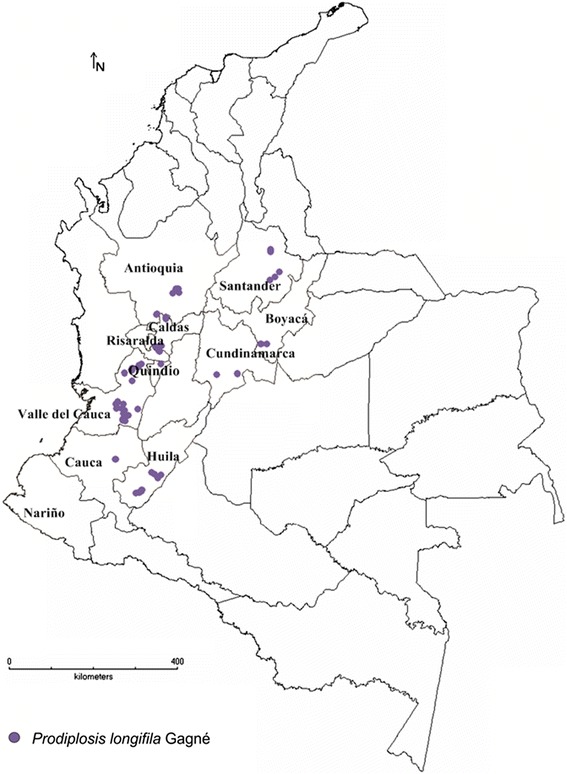
Map of the spatial distribution of *Prodiplosis longifila* in Colombia.

In total, 540 microscope slides were prepared; 485 corresponded to *P.longifila*, and the rest corresponded to other species of Cecidomyiidae. Table 1 and Figure 1 show the presence of *P. longifila* by department and sampled crops.

**Table 1:** **Composition of Cecidomyiidae species collected in 11 Colombian departments in crops of** ***Citrus*** ^**x**^ ***latifolia*** **(CL),** ***Solanum lycopersicum*** **(SL),** ***Capsicum frutescens*** **(CF),** ***Capsicum annuum*** **(CA),** ***Solanum tuberosum*** **(ST) and** ***Asparagus officinalis*** **(AO)**

**Crop**	**Organ**	**Department**	**# Samplings**	**Species**
CL	Flower buds	Antioquia	4	*Prodiplosis longifila*
				*Prodiplosis floricola*
		Caldas	1	*Prodiplosis longifila*
		Huila	3	*Prodiplosis longifila*
				*Prodiplosis floricola*
		Santander	1	*Prodiplosis longifila*
		Valle del Cauca	3	*Prodiplosis longifila*
				*Prodiplosis floricola*
SL	Leaves, flowers and fruits	Antioquia	18	*Prodiplosis longifila*
		Boyacá	12	*Prodiplosis longifila*
		Caldas	8	*Prodiplosis longifila*
		Cauca	4	*Prodiplosis longifila*
		Cundinamarca	6	*Prodiplosisl ongifila*
		Huila	12	*Prodiplosis longifila*
		Quindío	4	*Prodiplosis longifila*
		Risaralda	4	*Prodiplosis longifila*
		Santander	13	*Prodiplosis longifila*
		Valle del Cauca	26	*Prodiplosis longifila*
CF	Fruits	Valle del Cauca	10	Cecidomyiidae sp1
CA	Fruits	Antioquia	2	-
		Huila	6	*Prodiplosis longifila*
				Cecidomyiidae sp1
		Santander	1	*Prodiplosis longifila*
				Cecidomyiidae sp1
		Valle del Cauca	7	*Prodiplosis longifila*
				Cecidomyiidae sp1
ST	-	Antioquia	1	-
	-	Boyacá	15	-
	-	Nariño	5	-
AO	-	Cauca	1	-

**Figure 2:**
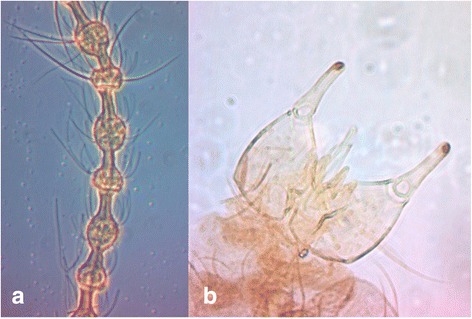
*Prodiplosis longifila* male (40X) **a**. antenna and **b**. genitalia.

*P. longifila* males have a soft and thin body and the R5 vein is slightly curved beyond the wing apex. The antennae have 14 flagellomeres, each consisting of two nodes (Figure 2a). The male gonopods are oriented dorsoventrally (Figure 2b).

### Characterization of damage in crops of tomato, pepper, paprika and Tahiti lime

#### Tomato (*Solanum lycopersicum*)

**Figure 3:**
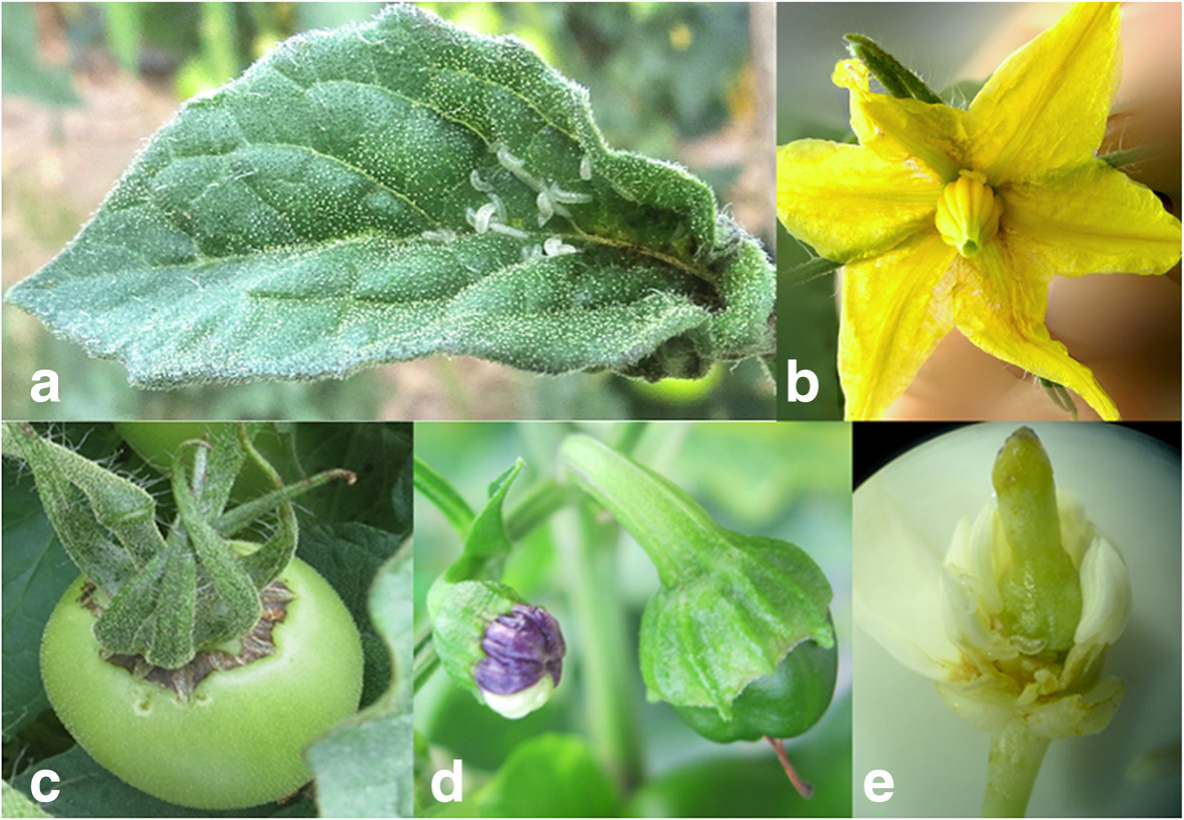
*Prodiplosis longifila* larvae damage in **a**. tomato leaf bud, **b**. tomato flower, **c**. tomato fruit, **d**. sweet pepper fruit, and **e**. Tahiti lime floral bud.

The female lays its eggs in leaf buds and flowers and under the calyx. When the larvae hatch, they feed by sucking the juices from the epidermal tissues of leaf buds (Figure 3a), flowers (ovaries and stamens, Figure 3b) and small fruits (Figure 3c); because of this, the tissues become brown only after the larvae drop to the soil. For example, in flowers, the stamens become brown, and later, the flower falls. The symptoms produced by *P. longifila* in flowers are very similar to those caused by *Botrytis cinerea* Pers. ex Fr. The fruit necrotises around the petiole, forming a spot known as “caregato” (in Spanish) or scab, and the fruit loses its commercial value (Figure 3a).

#### Sweet pepper (*Capsicum annuum*)

The larvae of *P. longifila* damage fruits. The larvae are white, and the small fruits (2 cm in length) that are affected changed from green to fuchsia colour and stop their growth (Figure 3d).

#### *Tahiti lime (Citrus*^x^*latifolia)*

*P. longifila* larvae were found in this crop consuming epidermal tissue of the ovaries, pistils and stamens of flower buds and flowers. In the ovary, necrosis was present after the larvae leave and after the abscission of flowers and small fruits. This study reports for the first time the presence of *P. longifila* feeding on flower buds of the Tahiti lime in Colombia (Figure 3e). Adults of *P. longifila* in the USA are morphologically similar to those of Colombia and, to those of tomato and sweet pepper.

### Actual and predicted geographical distribution of *P. longifila* in Colombia

**Figure 4:**
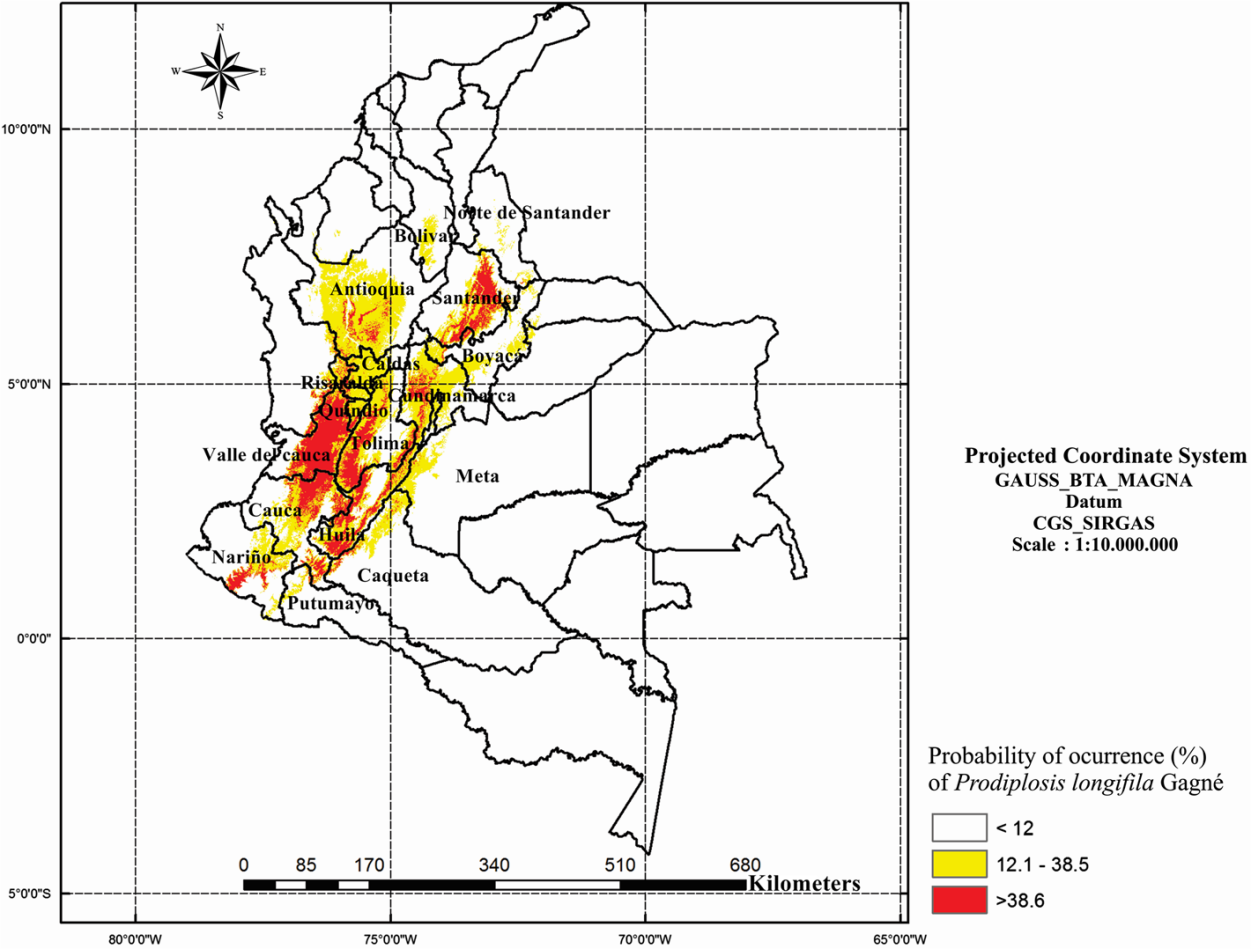
Predictive distribution map of *Prodiplosis longifila* in Colombia.

The insect was found between 739 and 2168 m.a.s.l. Figure 1 shows the distribution map of *P. longifila* in Colombia in tomato, sweet pepper and Tahiti lime. Regarding the predictive model on tomato, when analysing the correlation matrix of variables (Table 2), it was observed that the mean annual temperature (BIO_1) presents correlation coefficients close to 1 with the maximum temperature of the warmest month (BIO_5), the minimum temperature of the coldest month (BIO_6), the mean temperature of the wettest trimester (BIO_8) and the mean temperature of the driest trimester (BIO_9). The values of the variance inflation are presented in Table 2. The significant variables associated with the presence/absence of *P. longifila*, in order of most to least important, were: altitude, bio_4 (temperature seasonality), bio_10 (mean temperature of the warmest trimester), bio_11 (mean temperature of the coldest trimester), bio_19 (precipitation of the coldest trimester), bio_16 (precipitation of the wettest trimester) and bio_15 (precipitation seasonality). Altitude was the variable that had the most relevance. The map of the predictive model of the potential distribution of *P. longifila* in Colombia is presented in Figure 4. The AUC value for the training data was 0.968 and the accuracy of the predictive map was good with respect to the current distribution (kappa = 0.5).

**Table 2:** Matrix of correlations between climatic variables*, values of variance inflation (VIF) and Student’s ***t*** **test**

	**Bio1**	**Bio2**	**Bio4**	**Bio5**	**Bio6**	**Bio8**	**Bio9**	**Bio10**	**Bio11**	**Bio12**	**Bio13**	**Bio14**	**Bio15**	**Bio16**	**Bio17**	**Bio18**	**Bio19**	**VIF**	p**
**Bio1**	-																	13245.23	0.030
**Bio2**	0.05	-																35.27	0.261
**Bio4**	0.49	−0.46	-															5.60	0.024
**Bio5**	0.99	0.09	0.51	-														2045.72	0.244
**Bio6**	0.99	−0.04	0.51	0.98	-													1805.22	0.307
**Bio8**	0.99	0.06	0.48	0.99	0.99	-												733.62	0.268
**Bio9**	0.99	0.02	0.50	0.99	0.99	0.99	-											3304.18	0.284
**Bio10**	0.99	0.01	0.53	0.99	0.99	0.99	0.99	-										1.06	0.030
**Bio11**	−0.17	0.35	−0.12	−0.14	−0.23	−0.16	−0.19	−0.18	-									2.07	0.008
**Bio12**	0.20	−0.52	0.28	0.19	0.27	0.19	0.22	0.22	−0.47	-								7.98	0.482
**Bio13**	0.40	−0.62	0.61	0.19	0.46	0.38	0.43	0.43	−0.45	0.84	-							178.54	0.437
**Bio14**	−0.16	−0.29	−0.21	−0.2	−0.10	−0.17	−0.15	−0.17	−0.54	0.57	0.34	-						35.09	0.124
**Bio15**	0.49	−0.26	0.60	0.51	0.50	0.48	0.51	0.52	−0.06	0.34	0.63	−0.38	-					2.04	0.039
**Bio16**	0.41	−0.64	0.64	0.41	0.47	0.39	0.43	0.44	−0.42	0.82	0.99	0.30	0.62	-				1.52	0.049
**Bio17**	0.04	−0.51	0.08	0.01	0.11	0.03	0.06	0.05	−0.59	0.77	0.68	0.87	−0.06	0.65	-			68.87	0.096
**Bio18**	−0.33	−0.45	−0.21	−0.38	−0.27	−0.33	−0.32	−0.33	−0.37	0.47	0.30	0.71	−0.20	0.26	0.67	-		14.65	0.057
**Bio19**	0.47	−0.57	0.61	0.47	0.53	0.44	0.5	0.50	−0.41	0.78	0.97	0.27	0.67	0.97	0.61	0.18	-	3.23	0.018
**Altitude**																		5.98	0.002
**Temperature**																		4.81	0.415
**Relative Humidity**																		5.19	0.103

*Bio1: Mean annual temperature, **Bio2**: Mean diurnal temperature range (Max. Temp. – Min. Temp.), **Bio4**: Temperature seasonality (standard deviation x 100), **Bio5**: Maximum temperature of the warmest month, **Bio6**: Minimum temperature of the coldest month, **Bio8**: Mean temperature of the wettest trimester, **Bio9**: Mean temperature of the driest trimester, **Bio10**: Mean temperature of the hottest trimester, **Bio11**: Mean temperature of the coldest trimester, **Bio12**: Total annual precipitation, **Bio13**: Precipitation of the wettest month, **Bio14**: Precipitation of the driest month, **Bio15**: Precipitation seasonality (Coefficient of variation), **Bio16**: Precipitation of the wettest trimester, **Bio17**: Precipitation of the driest trimester, **Bio18**: Precipitation of the warmest trimester, **Bio19**: Precipitation the coldest trimester.

**Probability by Student’s *t* test.

Based on the distribution prediction map generated for *P. longifila*, an increase in the distribution of *P. longifila* to Nariño, Meta and Caquetá is predicted. The values with a higher probability of occurrence are in Huila, Tolima and Valle del Cauca, where tomato, Tahiti lime and sweet pepper are grown.

## Discussion

*P. longifila* feeds on tomato (leaf buds, flowers and fruits) and sweet pepper (small fruits). Here, it is reported for the first time consuming Tahiti lime in Colombia (flower buds). *P. longifila* damage is easily detected in tomato and sweet pepper, but in flower buds of Tahiti lime, it may be confused with fungal infections that can occur after larval feeding (Peña and Duncan 1992) or the spores can be transmitted by Cecidomyiidae adults (Mongrain et al. 2000). To avoid confusion, it is recommended to open the bud to check for the presence of larvae (Kikkert et al. 2006).

The identification of this new Colombian host indicates on one hand that *P. longifila* is polyphagous and not monophagous, like many other Cecidomyiidae (Hall et al. 2012), and that it adapts to phytochemically unrelated hosts. This adaptation probably occurs by a process of ecological speciation favouring alleles (Lenormand 2012) that allow adaptation to new and abundant hosts (Bourguet et al. 2014). The Tahiti lime, for example, was introduced in the Valle del Cauca (the zone where *P. longifila* was initially detected) in 1941 (Orduz and Mateus 2012) and *P. longifila* probably adapted to this host after coming from tomato that was being cultivated in the region. The propensity of this species for polyphagia may permit the expansion to other crops and areas of Colombia, as indicated by our risk map. Gagné and Jaschhof (2014) reported this species in five families and eight species of host plants. It is unknown if *P. longifila* causes economic damage in Tahiti lime in Colombia as it did in the United States, where it caused losses of up to 25% (Peña et al. 1987). Because Tahiti lime is exported from Colombia, the presence of *P. longifila* should be investigated further. On the other hand, the identification of *P. longifila* was carried out only by morphological characteristics and therefore DNA analysis is needed to understand if the populations of *P. longifila* collected from different host plants correspond to a complex of cryptic species rather than a single polyphagous species as suggested by Mathur et al. (2012) for the cecidomyiid *Dasineura oxycocanna.* Crops are usually introduced on purpose by humans and offer a new resource where a pest can evolve from pre-existing local herbivores by ecological speciation (Bourguet et al. 2014).

Although Kroschel et al. (2012) and Cisneros (1995) reported *P. longifila* as a key pest in asparagus, it was not found on this host, an absence that coincides with that reported for Colombia by Caicedo and Bellotti (2001).

In the Andean highland tropics (altitude >1000 m.a.s.l.), the altitude is inversely correlated with temperature. This could be the reason why the pest was not found in potatoes in Colombia, given that optimal production of that crop occurs between 2500 and 3000 m.a.s.l. in the Andes (Espinal et al. 2005), while in Peru, it is a pest on potatoes grown in low coastal areas (altitude <500 m.a.s.l.) (Kroschel et al. 2012). Similarly, low temperatures should reduce the development time of *P. longifila*, as it happens with the predatory midge *Feltiella acarisuga* (Vallot) (Gillespie et al. 2000). In contrast, warm regions favour the presence of *P. longifila* (e.g.,Andean valleys at approximately 1000 metres), in part because increasing temperature decreases the development time in some species of Cecidomyiidae (Baxendale et al. 1984), favouring population growth (Olfert et al. 2006).

Variables associated with precipitation also influence the distribution of *P. longifila*. The infestation of *P. longifila* in tomato decreases in times of high rainfall (Mena 2012), possibly because larvae drop from the foliage to the ground and because the high concentration of moisture and lack of oxygen in the soil will cause the death of the pupae (Yee 2013). However, after a period of drought, the combination of accumulated temperature and precipitation will favour the end of the pupal stage on the ground, stimulating adult emergence (Jacquemin et al. 2014). It is unknown whether *P. longifila* presents larval diapause similarly to *Contarinia nasturtii* Kiefferas an adaptation to unfavourable conditions of temperature and humidity (Readshaw 1966; Chen and Shelton 2007).

The results suggest that *P. longifila* is adapted to the hot and temperate climates of the Andean region (well established in the Valle del Cauca and the Coffee Region: Caldas, Risaralda and Quindío), and even in the departments at risk, as in Meta and Caquetá, the species would be on the eastern slope of the Andes. Because the foothills of Meta have 1000 ha cultivated with Tahiti lime (10% of the total Colombian citrus cultivation area) and are seen as an area to increase Tahiti lime production for exportation from Colombia (Cleves-Leguízamo et al. 2012), it is necessary to define whether *P. longifila* causes economic damage to this crop even if molecular analysis reveal the presence of cryptic species.

Although the model reflects the great expansion of *P. longifila* on tomato in Colombia since it was first reported in the Valle del Cauca and the Coffee Region, the model also represents the climatic conditions that potentially limit the distribution of the species in the absence of negative interactions with other species in what constitutes its fundamental niche (Parsa et al. 2012). Therefore, the risk map does not take into account the anthropogenic effect related to the marketing of fruits and seedlings and the limited effectiveness of quarantine measures (Pysck and Richardson 2010) that control the transfer of plant material, which contributes to the distribution of the species (Worner and Gevrey 2006). Agricultural practices such as the frequent application of insecticides promote infestations of *P. longifila* (Kroschel et al. 2012). Additionally, tomato crop irrigation changes the soil moisture, which could interfere with the development of pupae (Chen et al. 2011). The model does not include the effect of natural enemies such as species of the parasitoid *Synopeas* (Hymenoptera: Platygasteridae), which is responsible for the partial regulation of *P. longifila* in Tahiti lime in the USA (Peña et al. 1990) and is present in asparagus infested with *P. longifila* (Cisneros 1995). *P. longifila* is parasitized by several species of *Synopeas* in Colombia (unpublished data by the authors), which could influence its distribution.

The predictions of our model call for an increase in preventive measures, such as monitoring the insect presence in nurseries from where *P. longifila* larvae could spread (Gagné 1986b; Sylven and Lövgren 1995). Unpublished data of the authors indicate that the development time of *P. longifila* in tomato and Tahiti lime fluctuates between 9 and 14 days, which would allow the relatively rapid establishment of the species in the areas of risk.

## Conclusions

This study provides information on the biology of the gall midge *Prodiplosis longifila,* one of the most destructive pests of tomato and paprika in Colombia. *P. longifila* is reported for the first time attacking reproductive structures of Tahiti lime in Colombia. Microscopic preparations of *P. longifila* males are needed for taxonomic identification; the antennae have 14 flagellomeres with two nodes each, and the male gonopods are oriented dorsoventrally. The insect is distributed between Andean valleys located at 739 up to 2168 masl in The Andes of Colombia. Apparently, unchecked transport of plant material has contributed to the dispersion of the species, so sanitation measures should be implemented in nurseries. Pruning tomato leaves may reduce the population of *P. longifila* larvae. Besides altitude, the variables that most influence the distribution of *P. longifila* are temperature, relative humidity and precipitation. The infestation of *P. longifila* in tomato decreases during periods of high rainfall, possibly because larvae drop from the foliage to the ground, but also because the high concentration of moisture and lack of oxygen in the soil may increase pupal mortality. Prediction models based on tomato crop populations predict the distribution of the species on the foothills of Meta area, located on the eastern slope of the Andes, where 10% of total Colombian citrus cultivation takes place. Some years ago *P. longifila* caused economic losses to Tahiti lime production in the USA, therefore it is important to further investigate its impact on Tahiti lime production in Colombia. However DNA analysis for *P. longifila* is required to understand if the populations of *P. longifila* collected from different host plants correspond to a complex of cryptic species rather than a single polyphagous species. These results will be fundamental to develop effective IPM strategies, including biological control programmes.

## Methods

### Study site

Between March 2012 and September 2013, crops of peppers, asparagus, Tahiti lime, potato, paprika and tomato were sampled in the departments of Antioquia, Boyacá, Caldas, Cauca, Cundinamarca, Huila, Nariño, Quindío, Risaralda, Santander and Valle del Cauca in Colombia in search of larvae of *P. longifila*. Leaf buds, flowers and fruits in early stages of development that showed signs of damage in tomato were inspected for larvae (Mena 2012); the leaves, flowers and fruits with symptoms of damage in pepper and paprika were also inspected. In asparagus, young buds were searched for larvae, while they were sought in the foliage, flowers and fruits of potatoes (Kroschel et al. 2012) and the flower buds of Tahiti limes (Peña et al. 1987). The discoloration and shape of the affected structures were described and photographed to characterize the damage.

### Taxonomic identification of *P. longifila*

Plant structures that had damage and/or larvae were removed, packed in plastic containers (20 × 10 cm) with wet paper towels to prevent drying and transported to the Laboratory of Entomology and Acarology of the National University of Colombia at Palmira. To obtain adults, recovery chambers were used, as described by Gagné (1994), and kept in an acclimated chamber (Panasonic MLR-351) under controlled conditions (23°C, 75% HR, 12 L:12O). Upon reaching full development, the adults were stored in 95% ethyl alcohol.

Taxonomic identification was based on the morphology of males (Gagné 1986a; Gagné 1994); the abdomen, antennae and wings were removed from each individual, and the rest of the body was kept for further molecular identification. With the removed structures, microscope slides were made, according to the protocol of Gagné (1994). Adult *P. longifila* were collected in Tahiti lime of Florida (USA) and sent by JE Peña (University of Florida); these samples were also processed for identification.

### Distribution model of *P. longifila*

To build the current distribution map of *P. longifila*, georeferenced points identified with a GPS (Garmin GPSMAP 60CSx) were mapped using the DIVA-GIS program.

The ecological niche model (Peterson 2003; Warren and Seifert 2011) was used to predict the distribution of *P. longifila* in tomato in Colombia. To build the model, 90 sampling points corresponding to tomato crops were used, and for its validation, 39 points were used. At each sampling point, the altitude was determined. Climatic data obtained from the WorldClim database (http://www.worldclim.org, Hijmans et al. 2005) were used for the construction of the environmental layers of the ecological niche model. The database has 19 variables derived from temperature and precipitation.

Variables with more influence on the distribution of *P. longifila* were selected. This selection was done by using a multi co-linearity test to determine whether there was a correlation between the independent variables. This could inflate both the standard error and the confidence intervals and could prevent the determination of the significance of each variable on the dependent variable (Quinn and Keough 2002). Therefore, a Pearson correlation coefficient matrix was constructed with the 22 recorded variables (19 WorldClim variables plus 3 field variables). Variables with R ≥ 0.80 were considered to be correlated (Lozier and Mills 2009). The tolerance value (1-r2) for each variable was then checked and expressed as a factor of overestimation of the variance (VIF = (1-r2) -1). A low tolerance indicates that the variable is correlated with one or more variables; therefore, VIF values above ten suggest a strong collinearity (Quinn and Keough 2002). Variables with VIF values >10 were eliminated. Finally, the association between the remaining variables and the dependent variable (the presence/absence of *P. longifila*) was evaluated. To do this, a Pearson correlation was performed, and its significance was estimated by Student’s t test. Once the variables with the greatest effect on *P. longifila* were selected, MAXENT 3.3.3 k (Maximum Entropy, Elith et al. 2011) was used to construct the ecological niche model. This software generates an estimate of the probability of occurrence of the species as a function of the selected environmental variables (Phillips et al. 2006). The results from this program were processed in ArcGIS 9.3.

The Jackknife Maxent test was used to determine the importance of each variable and its relationship with *P. longifila*. To evaluate the model performance, the AUC (area under the curve) value, which is an independent measure of the threshold model performance, was used; AUC = 1.0 optimum; AUC = 0.5 weak (Araújo et al. 2005). The model was validated with Cohen’s Kappa (Fielding and Bell 1997); if 0.4 < Kappa <0.75, the model is considered good (Fielding and Bell 1997).
